# SpotOn: High Accuracy Identification of Protein-Protein Interface Hot-Spots

**DOI:** 10.1038/s41598-017-08321-2

**Published:** 2017-08-14

**Authors:** Irina S. Moreira, Panagiotis I. Koukos, Rita Melo, Jose G. Almeida, Antonio J. Preto, Joerg Schaarschmidt, Mikael Trellet, Zeynep H. Gümüş, Joaquim Costa, Alexandre M. J. J. Bonvin

**Affiliations:** 1 CNC - Center for Neuroscience and Cell Biology; Rua Larga, FMUC, Polo I, 1°andar, Universidade de Coimbra, 3004-517 Coimbra, Portugal; 2 Bijvoet Center for Biomolecular Research, Faculty of Science - Chemistry, Utrecht University, Utrecht, 3584CH The Netherlands; 3 Centro de Ciências e Tecnologias Nucleares, Instituto Superior Técnico, Universidade de Lisboa, Estrada Nacional 10 (ao km 139,7), 2695-066 Bobadela LRS, Portugal; 4 Department of Genetics and Genomics and Icahn Institute for Genomics and Multiscale Biology, Icahn School of Medicine at Mount Sinai, New York, NY USA; 5 CMUP/FCUP, Centro de Matemática da Universidade do Porto, Faculdade de Ciências, Rua do Campo Alegre, 4169-007 Porto, Portugal

## Abstract

We present SpotOn, a web server to identify and classify interfacial residues as Hot-Spots (HS) and Null-Spots (NS). SpotON implements a robust algorithm with a demonstrated accuracy of 0.95 and sensitivity of 0.98 on an independent test set. The predictor was developed using an ensemble machine learning approach with up-sampling of the minor class. It was trained on 53 complexes using various features, based on both protein 3D structure and sequence. The SpotOn web interface is freely available at: http://milou.science.uu.nl/services/SPOTON/.

## Introduction

The human interactome consists of more than 400,000 protein-protein interactions (PPIs), which are fundamental for a wide-range of biological pathways^1–3^. Adding the structural dimension to the interactome is crucial for gaining a comprehensive understanding at atomic level of molecular function in human diseases^4^. Furthermore, accurate identification of key residues participating in PPIs is critical to understand disease-associated mutations and fine-tune PPIs. Achieving this paves the way to the development of new approaches and drugs to modulate those interactions^4, 5^. Critical for the understanding of PPIs has been the discovery that the driving forces for protein coupling are not evenly distributed across their interaction surfaces. Instead, typically, a small set of residues contributes the most to binding, the so-called binding Hot-Spots (HS). A well accepted definition for HS residues are those which, upon alanine mutation, generate a binding free energy difference (ΔΔG_binding_) ≥2.0 kcal/mol. Conversely, Null-spots (NS) correspond to residues with ΔΔG_binding_ <2.0 kcal/mol when mutated to alanine^4^.

HS identification through experimental approaches is based on molecular biology methods which provide accurate results. However, these techniques are complex, time-consuming and expensive. The necessity of expressing and purifying each individual protein before measurement leads to the low-throughput of these techniques, which is a major bottleneck in HS identification^6^. Hence, computational approaches for HS prediction can render a viable alternative to experimental techniques, providing valuable insight and high-throughput HS identification. Statistical and Machine-Learning-based (ML) methods are highly attractive approaches for computational biology as they can be applied in a large scale manner at relatively low computational costs^7, 8^. Computational ML approaches to HS prediction tend to fall into two broad categories: i) sequence-based methods which use an encoding of sequence-derived features of the residues and their neighbours and then explore amino-acid identity, physicochemical properties of amino-acids, predicted solvent accessibility, Position-Specific Scoring Matrices (PSSMs), conservation in evolution and interface propensities; and ii) structure-based methods that use an encoding of structure-based features of the target residues and neighbours such as propensities at interface and surface, interface size, geometry, chemical composition, roughness, SASA, atomic interactions, among others^1–10^. Furthermore, both categories can be combined in some methods^8^. A detailed review of current ML algorithms applied to HS detection can be found in Moreira’s review^3^.

According to a recent comprehensive review^9^ and demonstrated by a series of recent publications^10–12^ to establish a really useful computational tool for a biological system, we need to consider the following procedures: (i) construct or select a valid benchmark dataset to train and test the model; (ii) formulate the biological samples with an effective mathematical expression that can truly reflect their intrinsic correlation with the target to be analyzed; (iii) introduce or develop a powerful algorithm (or engine) to operate the analysis; (iv) properly perform cross-validation tests to objectively evaluate the anticipated accuracy of the statistical method and (v) establish a user-friendly web-server for the method that is accessible to the public.

Here, we describe a new HS predictor implemented as a freely accessible web portal. For the past several years, we have been developing new tools and methodologies to accurately predict HS. Our first predictor was trained on 13 features^13^, which was subsequently extended to 75 in a more recent work^8, 14^. The database used in this work includes 53 non-redundant protein complexes with alanine scanning mutagenesis data, genetic conservation scores and three dimensional (3D) crystallographic structures, comprising a total of 534 mutations. It was derived from the Alanine Scanning Energetics database (ASEdb)^15^, the Binding Interface Database (BID)^16^, the Protein-protein Interaction Thermodynamic (PINT)^17^ and the Structural database of kinetics and energetic of mutant protein interactions (SKEMPI)^18^. We have considered and computed over 880 features, evaluated 51 classifiers, and compared their performance in 6 different pre-processing sets. These classifiers were subjected to hierarchical clustering and grouped in 5 different clusters. The algorithms’ performance in each cluster was compared and the best one was selected for the creation of an ensemble approach by logistic regression. The final method shows a F1-score 0.97, the highest accuracy reported in the literature so far for HS prediction. The predictor is implemented in a new and user-friendly web-server, “SpotOn” (Hot SPOTs ON protein complexes), which is freely available at: http://milou.science.uu.nl/services/SPOTON/.

## Results

### Features for Hot-Spot prediction

The accuracy of ML depends largely on the quality of the feature sets and the experimental data available to train the model. From the few databases containing information about experimentally determined HS, a non-redundant representative dataset can be constructed with a vast coverage of all relevant type of interactions. However, these data, as the majority of data in biology, are still atypical for ML: they are too sparse and incomplete, too biased and too noisy^19^. Moreover, the field is marked by imbalanced data, which renders the selection of proper performance measures and algorithms even more important.

The dataset used in this work includes 534 residues from 53 protein-protein complexes (127 HS and 407 NS), which were divided into training and test sets (see Methods). For these, we calculated 881 features, 35 structure-based features and the remaining evolutionary/sequence-based. From a structural perspective, the focus is on the Solvent Accessible Surface Area (SASA), the type of residues at the binding interface and the intermolecular interactions established. We also introduced PSSM and five different types of sequence characterization (proportion of each amino-acid type, pseudoamino-acid composition, BLOSUM, protein fingerprinting and proteochemometric modelling). Since raw data usually show a high variability for various features, we first converted all features in the training set into z-scores (i.e. each feature has its mean subtracted and is divided by its standard deviation). The same procedure was performed on the testing set, but using the mean and standard deviation derived from the training set instead. This is essential as it provides a better estimation of the quality and scalability of our model. Principal Component Analysis (PCA), a technique which works by orthogonally transforming the data to convert a set of highly correlated features into a set of linearly uncorrelated ones, principal components, was also applied to our dataset in a different pre-processing condition, to tackle the high dimensionality problem. PCA was chosen as it offers an acceptable trade-off between computational time, data variance and model performance^20^. We choose the principal components that account for a cumulative percentage variance $\frac{{\sum }_{i=1}^{d}{\lambda }_{i}}{{\sum }_{i}{\lambda }_{i}}\ge 95 \%$. Different datasets were thus created:

i) Scaled - dataset generated upon centering and scaling of variables;
ii) ScaledUp - dataset generated upon centering and scaling of variables and up-sampling of the minor class (HS);
iii) ScaledDown - dataset generated upon centering and scaling of variables and down-sampling of the major class (NS);
iv) PCA - dataset generated upon centering and scaling of variables and PCA;
v) PCAUp - dataset generated upon centering, scaling and PCA of variables and up-sampling of the minor class (HS);
vi) PCADown - dataset generated upon centering, scaling and PCA of variables and down-sampling of the major class (NS).

### Machine Learning Algorithms Clustering

**Figure 1:**
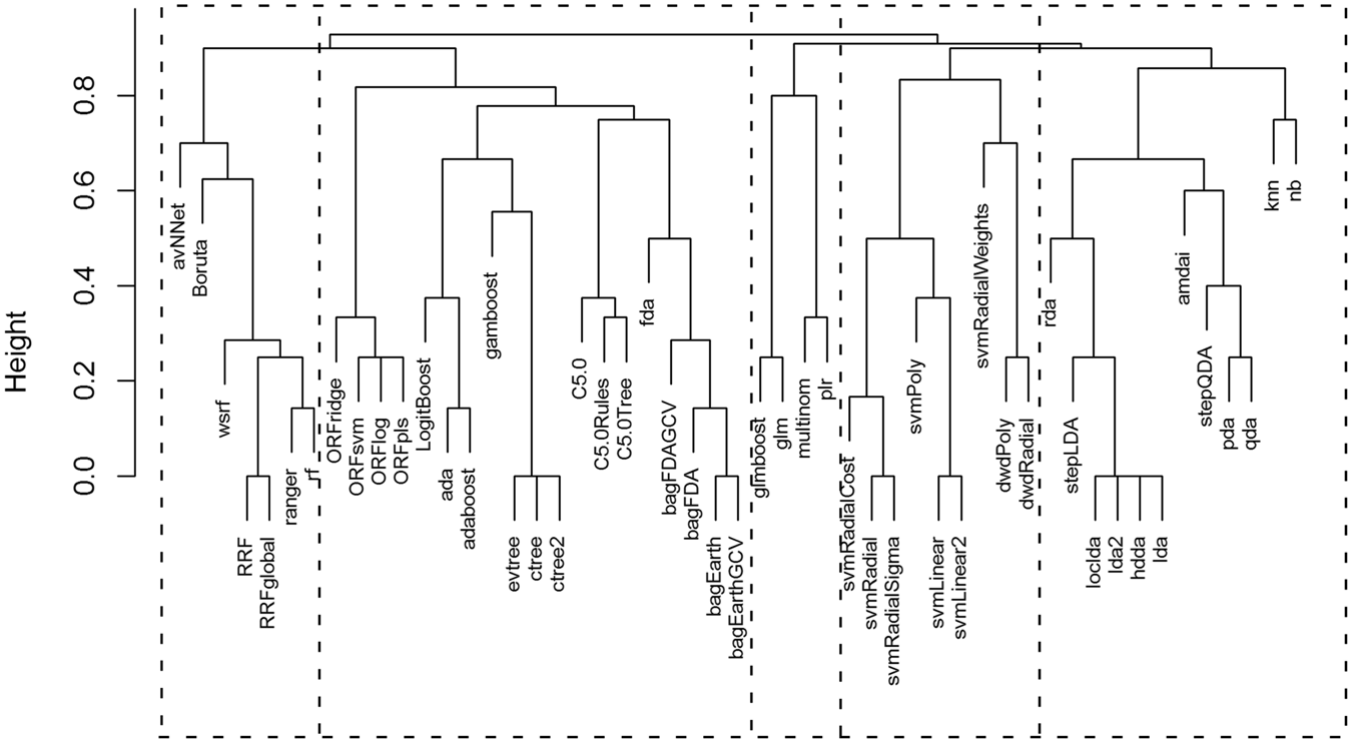
Cluster dendrogram of the machine learning algorithms tested in this work. All 5 clusters are separated by a dashed line and are ordered from I to V.

51 algorithms were tested and their performance was evaluated through a myriad of statistical metrics (fully described in the Methods section). For a better performance comparison, and due to the difficulty in categorizing ML approaches in a simple way, we began by characterizing them in agreement with Caret’s tags^21^ as binary attributes – 1 or 0, based on the presence or absence of that tag, respectively. The various ML algorithms were then subjected to hierarchical clustering, which returned a distance matrix based on the Jaccard similarity coefficient as a metric and the complete aggregation scheme. The dendrogram depicted in Fig. 1, allows us to distinguish 5 main algorithm clusters:

I) Cluster I (mostly tree-based models): avNNet, Boruta, ranger, rf, RRF, RRFglobal and wrsf;
II) Cluster II (mostly adaptive algorithms, bagging algorithms and decision trees/forests): ada, adaboost, bagEarth, bagEarthGCV, bagFDA, bagFDAGCV, C5.0, C5.0Rules, C5.0Tree, ctree, ctree2, evtree, fda, gamboost, LogitBoost, ORFlog, ORFpls, ORFridge and ORFsvm;
III) Cluster III (mostly regression models): glmboost, multinom, glm and plr.
IV) Cluster IV (mostly support vector machines and distance weighted algorithms): dwdPoly, dwdRadial, svmLinear, svmLinear2, svmPoly, svmRadial, svmRadialCost, svmRadialSigma and svmRadialWeights;
V) Cluster V (mostly discriminant analysis algorithms): amdai, hdda, knn, lda, lda2, loclda, nb, pda, qda, rda, stepLDA and stepQDA;

### ML algorithms Cluster Performance

Extensive statistical measures for the six datasets listed above that cover all possible aspects of the assessment proposed so far are provided in Annexes Tables SI-1 to SI-6. Algorithms that did not converge are not listed in those Annexes. Figure SI-1 illustrate the mean values and box-plot distributions of the sum of the Area under Receiver Operating Characteristic (AUROC), True Positive Rate (TPR) and True Negative Rate (TNR) metrics for all six datasets (pre-processing conditions) studied. For all, mostly Cluster I and Cluster II methods achieved peak performance, while Cluster IV and V were generally responsible for the worst scores. We performed various statistical analyses to access the real discrimination power between the 5 clusters of methods using one-way Multivariate Analysis of Variance (MANOVA) for all 6 datasets. The corresponding p-values are listed in Table SI-7. MANOVA is a parametric test that has some assumptions: multivariate normality of the data, multivariate homoscedasticity, no multicollinearity, and the absence of multivariate outliers. As all algorithms are organized already by similarity, they are not independent and these assumptions are not fulfilled by our data. Still, MANOVA is usually resistant upon violation of these assumptions, which means that we can statistically accept the attained results confidently. At a significance level of 0.05, MANOVA allows us to conclude that the 5 clusters perform differently for this dataset. The p-values for the MANOVA obtained for all 6 datasets were below 0.050 (PCA: 0.003; PCAUp: 0.001; PCADown: 0.0001; Scaled: 0.004; ScaledUp: 0.020; ScaledDown: 0.004), which allows us to conclude that the clustering process is discriminatory.

Table 1 summarizes the performance on the independent test set by presenting the mean values for each metric for the best classifier of each cluster for the different pre-processing conditions. More detailed information (best algorithm per cluster and its respective metrics) is provided in Table SI-8, while a visual representation of ROC curves for the best algorithms in the best pre-processing condition (ScaledUp) can be found in Fig. SI-2, accompanied by a paired bar plot showing sensitivity and specificity values for these algorithms. From the various pre-processed datasets described above, the ScaledUp (dataset generated upon centering and scaling of variables and up-sampling of the minor class) was subsequently used since it yielded the best performance metrics, specifically the best mean value for AUROC and TPR (Sensitivity) in the training set.

**Table 1:** Statistical metrics mean values attained from the best algorithms of each cluster for all pre-processing conditions for both training set (Train) and testing set (Test).

	Train	Test	Train	Test
	PCA		Scaled	
AUROC	0.79	0.67	0.80	0.77
Accuracy	0.89	0.78	0.90	0.81
Sensitivity	0.60	0.31	0.67	0.40
Specificity	0.98	0.92	0.97	0.94
PPV	0.87	0.53	0.88	0.67
NPV	0.89	0.81	0.91	0.83
F1-score	0.67	0.38	0.75	0.49
MCC	0.68	0.29	0.71	0.42
	**PCAUp**		**ScaledUp**	
AUROC	0.93	0.80	0.94	0.83
Accuracy	0.93	0.79	0.97	0.79
Sensitivity	0.95	0.55	0.98	0.48
Specificity	0.93	0.86	0.96	0.88
PPV	0.93	0.57	0.96	0.57
NPV	0.94	0.87	0.98	0.85
F1-score	0.94	0.55	0.97	0.52
MCC	0.83	0.41	0.91	0.38
	**PCADown**		**ScaledDown**	
AUROC	0.79	0.70	0.81	0.74
Accuracy	0.91	0.75	0.90	0.76
Sensitivity	0.90	0.78	0.87	0.66
Specificity	0.92	0.74	0.93	0.80
PPV	0.92	0.48	0.92	0.51
NPV	0.91	0.92	0.89	0.88
F1-score	0.91	0.59	0.89	0.57
MCC	0.78	0.46	0.78	0.42

PCA: dataset upon Principal Component Analysis; PCAUp: dataset upon Principal Component Analysis and up-scaling of the minor class; PCADown: dataset upon Principal Component Analysis and down-sampling of the major class; Scaled: dataset upon z-score calculation; ScaledUp: dataset upon z-score calculation and up-sampling of the minor class; ScaledDown: dataset upon z-score calculation and down-sampling of the major class.

Ensembles of machine-learning algorithms have shown to be quite valuable in improving classification when constructing ML models^22^. The best algorithms of each cluster for the ScaledUp pre-processing condition (ORFsvm, pda, rf, svmPoly and plr) were used as input for a logistic regression model. A stepwise selection of relevant variables (algorithms) was performed, leading to the selection of rf, svmPoly and pda as the most relevant classifications for the logistic regression model. Training and testing metrics are provided in Table 2. Logistic regression leads to improved results as reported by all metrics, for both the full (5 variable) and rf + svmPoly + pda regression models. Even though both share practically identical metrics, we chose the latter as our final model, since it offers the best possible predictions in the least time and simplest way when compared with the Full Regression model.

**Table 2:** Statistical metrics for the best algorithm of each cluster of method and their combined regression model, both the “Full Regression” and the stepwise-optimized regression model (rf + svmPoly + pda) for both training and testing set.

	C5.0		pda		plr		rf		svmPoly		Full Regression		rf + svmPoly + pda	
	Train	Test	Train	Test	Train	Test	Train	Test	Train	Test	Train	Test	Train	Test
AUROC	0.88	0.83	0.85	0.84	0.83	0.85	0.93	0.83	0.89	0.83	0.91	0.91	0.91	0.91
Accuracy	0.88	0.91	0.85	0.88	0.83	0.85	0.93	0.90	0.89	0.90	0.94	0.95	0.94	0.95
Sensitivity	0.78	0.68	0.86	0.76	0.82	0.84	0.87	0.71	0.80	0.68	0.98	0.98	0.98	0.98
Specificity	0.98	0.98	0.84	0.91	0.85	0.85	0.98	0.96	0.98	0.97	0.84	0.85	0.84	0.85
PPV	0.98	0.90	0.84	0.73	0.84	0.64	0.98	0.84	0.97	0.87	0.95	0.95	0.95	0.95
NPV	0.81	0.91	0.85	0.93	0.82	0.95	0.89	0.91	0.83	0.91	0.91	0.94	0.91	0.94
FPR	0.22	0.32	0.14	0.24	0.18	0.16	0.13	0.29	0.20	0.32	0.02	0.02	0.02	0.02
FNR	0.02	0.02	0.16	0.09	0.15	0.15	0.02	0.04	0.02	0.03	0.16	0.15	0.16	0.15
F1	0.86	0.78	0.85	0.74	0.83	0.73	0.92	0.77	0.88	0.76	0.96	0.97	0.96	0.97

PCA: dataset upon Principal Component Analysis; PCAUp: dataset upon Principal Component Analysis and up-scaling of the minor class; PCADown: dataset upon Principal Component Analysis and down-sampling of the major class; Scaled: dataset upon z-score calculation; ScaledUp: dataset upon z-score calculation and up-sampling of the minor class; ScaledDown: dataset upon z-score calculation and down-sampling of the major class.

In order to further assess the quality of our method, we compared it with other methods commonly used to perform HS prediction, namely SBHD2 (SASA-Based Hot-spot Detection)^14^ (a previous version of the algorithm considering only SASA-related features), Robetta^23^, K-FADE and K-CON models (KFC2-A and KFC2-B)^24^, and CPORT (Consensus Prediction Of interface Residues in Transient complexes)^25^, even though the latter is not a proper HS predictor but rather an interface predictor. All predictions were collected by using the respective web-servers. The performance of all tested methods is summarized in Table 3. Our full dataset was used for the comparison since it is the richest nonredundant database of proteins with resolved structure and information on HS. SpotOn clearly outperforms all other methods, with a strong performance in identifying both HS and NS.

**Table 3:** Comparison of the performance of SpotOn with other common methods used for HS prediction for the full dataset.

	SpotOn	SBHD2^13^	Robetta^23^	KFC2-A^24^	KFC2-B	CPORT^25^
AUROC	0.91	0.69	0.62	0.66	0.67	0.54
Sensitivity	0.98	0.70	0.29	0.53	0.28	0.54
Specificity	0.84	0.71	0.88	0.81	0.96	0.47
F1-score	0.96	0.62	0.39	0.56	0.42	0.42

### SpotON web-server implementation

#### Input

A screenshot of the submission page can be seen in Fig. SI-3. The interface requires the user to upload a 3D structure of the protein-protein complex in Protein Data Bank (PDB) format and specify the chain identifiers of the two monomers. The order in which the two proteins are provided is arbitrary. Instructions are available in the Help section of the server, in addition to popups in the submission page. Before submitting a run, users should register with an email address of their choice. Although the server is freely available, registration is required since the user email is used for various notifications about the progress of the job which might take typically between 30 and 90 minutes to complete, depending on the size of the complex and the server load.

### Output and representation of the results

**Figure 2:**
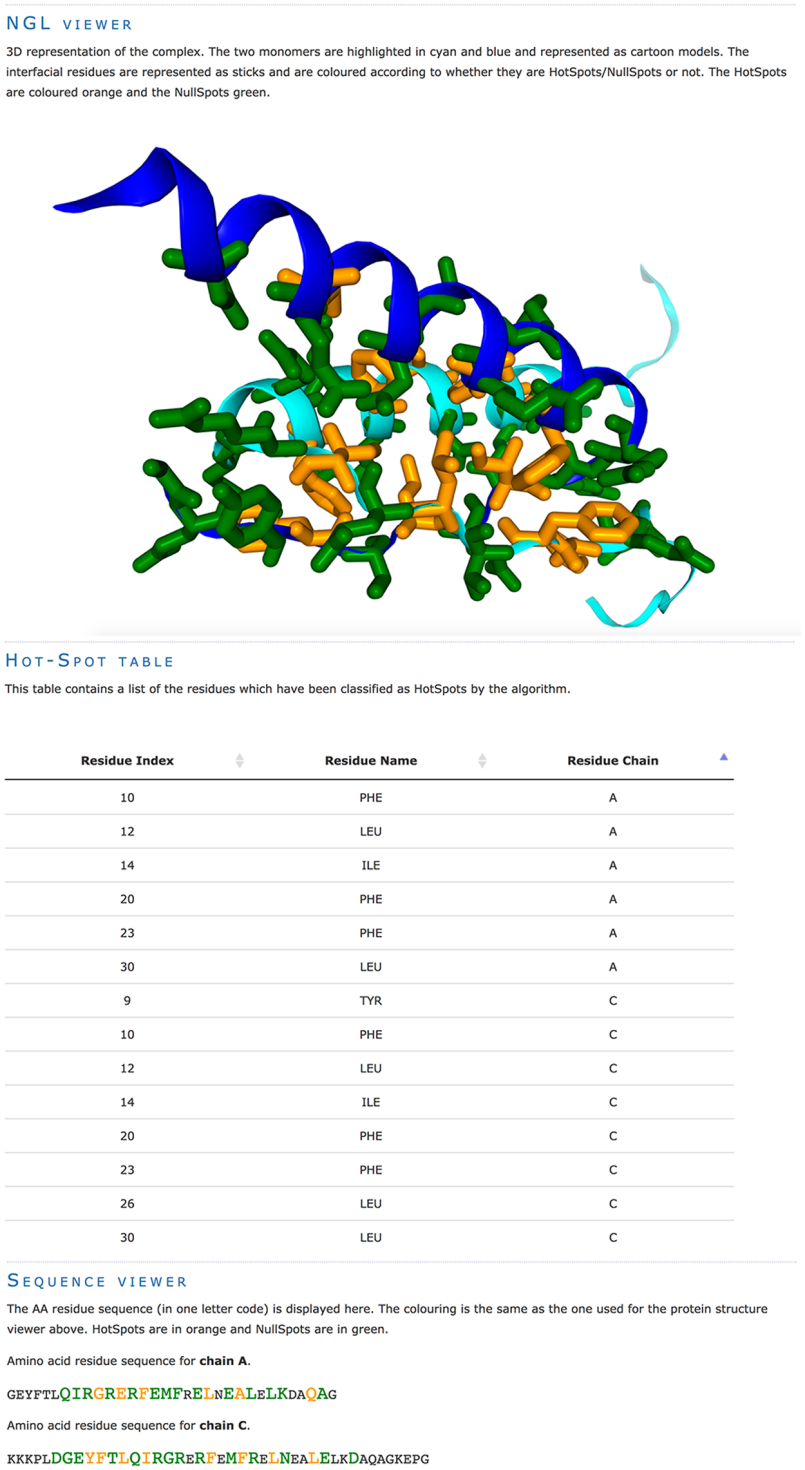
Collage of the results page of the SpotOn webserver: Screenshot of the webGL structure viewer highlighting the hot spot residues in the interface (top); table listing the residues classified as HS (middle) and; sequence viewer highlighting the residues classified as HS and NS in the full sequence of the chains submitted for analysis (bottom).

Upon successful job submission, users receive an email with the URL address where the output of the run will appear as soon as the analysis is complete. An additional email notification containing the URL of the results page is sent upon completion, informing users of the success or failure of their run. The main outputs of the server are two tables that list the residues classified as HS and NS. Figure 2 illustrates an output example for PDBid 3SAK^26^ and contains the list of residues predicted as HS. Any column can be used to sort the table. These tables are also made available as CSV files in the archive of the run that the user can download. The information is also visualized in the form of a sequence plot (Fig. 2), which enables users to quickly identify HS residues. Finally, the result page provides a direct visualization of the identified HS within the interface of the complex in the form of a webGL powered 3D structure viewer^27^ (Fig. 2).

For each run, all generated results are provided as a gzipped archive, which can be downloaded. It contains a CSV file that details all the calculated features for the interfacial residues, and the CSV files of the two tables shown on the results page.

### Implementation

**Figure 3:**
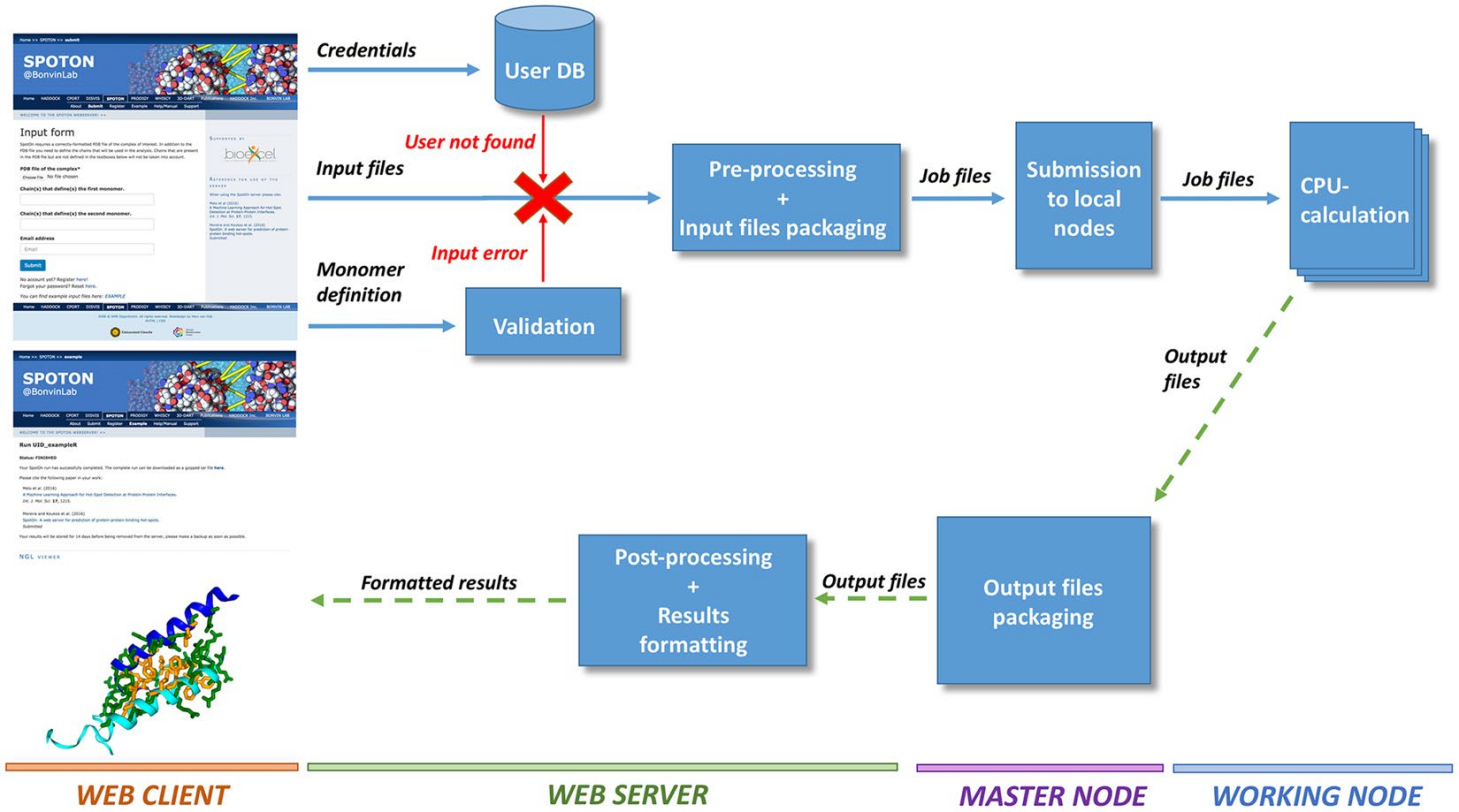
Workflow of the SpotOn web server pipeline. Each box corresponds to a step in the pipeline and the horizontal bars at the bottom of the image indicate the environment in which this step takes place. At the very beginning, the user is required to upload the PDB file in addition to defining the two monomers of the interface. After the credentials of the user have been checked and the input data validated, the web server will generate the run directory with all the necessary files. In case of validation errors, a helpful message is displayed on screen indicating the exact problem. The master node of the Linux cluster where SpotOn is hosted monitors the directory where the run folders are located and if the global maximum number of concurrent SpotOn jobs or the maximum number of jobs per user have not exceeded the defined limits, the analysis is submitted to the queue. Depending on the load of the system at the time of submission, the analysis might start running immediately or with a small delay. The user is notified as soon as the job starts running. The actual run takes place in one of the working nodes of the cluster and, upon completion, the user is notified via email.

The SpotOn server runs alongside other servers of our group (available at http://milou.science.uu.nl/) on a local Linux cluster. The backend is implemented in Python and R, but also makes use of external programs, including Visual Molecular Dynamics (VMD)^28^ and BLAST^29^ for the analysis. It makes use of the Flask microframework for web development in addition to the standard languages of the web (HTML, CSS, JS). Documentation is kept up-to-date and support is offered via spoton.csbserver@gmail.com and the BioExcel support forum (http://ask.bioexcel.eu). Calculations submitted by users are anonymous runs on separate directories with randomly generated 12-character key names. Results are kept on the server for 2 weeks. The server workflow is illustrated in Fig. 3. If any errors occur at any point of the pipeline illustrated in this figure the analysis will be terminated and an email will be sent to the users prompting them to review the output of the program. Submissions from users are processed in parallel with a maximum number of 15 jobs running simultaneously. Each user is limited to 3 concurrent runs.

## Discussion

In recent years ML has been proven to be crucial to unravel aspects of protein function from a vast majority of biomolecular data resources and it has become highly valuable in a myriad of areas for being a fast, inexpensive and high-throughput tool. This study focuses on a specific problem, the detection of HS, for which several machine-learning techniques have been developed^1–10^. Dataset selection and treatment as well as performance estimation are still major challenges in the application of ML to this field. To propose a general methodology, it is necessary to compare the performance of various algorithms and different data extraction techniques. Some classifiers (linear discriminant analysis or generalized linear models) come from statistics, others come from data mining (tree-based), and some are connectionist approaches (such as neural networks). All can behave differently when applied to different datasets. So, identifying the best classifier for a given problem is crucial, as the No-Free-Lunch Theorem from Wolpert^30^ states: “*The best classifier may not be the same for all the datasets*”. In this work, structure- and sequence-based features were combined to evaluate 51 classifiers and compare their performance on six differently pre-processed datasets. These classifiers were subjected to hierarchical clustering and grouped into 5 different clusters. We have compared the algorithms’ performance in each cluster and chosen the best of each for a global comparison. Within Cluster I, the top performance methods are either based on neuronal networks (avNNet) or on random forests (rf, RRFglobal). While avNNet, a simple shallow neural network, and rf, a forest composed of decision trees, are somewhat simple methods, RRFglobal is a regularized version of a basic random forest, capable of selecting the best feature subset with higher accuracy. Within Cluster II, the best methods are either bagging (bagEarth and bagEarthSVM), support vector machines-based (ORFsvm) or additive logistic regression models (ada). Bagging (bootstrap aggregating) generates several training subsets out of the original training set and performs a majority vote of all models. ORFsvm uses oblique decision trees which can split the feature space obliquely instead of using solely axis-parallel feature space splitting enabling a finer tuning of the model, which s explain their success. Ada uses boosting, creating an ensemble of logistic regression models, and therefore a stronger classification predictor. For Cluster III, the best results are achieved for regression models (glmboost and plr). Even though both are based on regression models, the key aspects of each is quite different as glmboost uses boosting to create an ensemble of generalized linear models, while plr uses L2 penalized regression models. L2 penalization is usually successful thanks to its ability to prevent overfitting by minimizing regression coefficients. Cluster IV is composed solely of SVM approaches. The most successful was svmPoly, which uses polynomial kernels of the original variables to construct a SVM, enabling it to act as a non-linear model. The other SVM, which was the best only in the PCA pre-sampling condition (with far worst F1-score, however), combines cost regularization that enables control over the smoothness of the fitted function, and a radial basis function that represents the input space as the distance between each vector. Cluster V features only discriminant analysis models (rda, amdai, pda and stepLDA) able to perform combinations of features for classification. Rda uses regularization to determine the best linear combination of features and fine tune their coefficients while amdai is essentially a regular discriminant predictor with slight alterations that render it capable of adapting to new classes in the testing set. Pda is a parametric discriminant classifier, which assumes a probability distribution for the population and stepLDA is a linear discriminant analysis featuring stepwise feature selection.

The clustering of the various ML algorithms by their common characteristics allowed us to combine their results into a ML ensemble that uses rf, svmPoly and pda. Our predictor outperforms the currently available methods in the literature with an AUROC of 0.91, sensitivity of 0.98 and specificity of 0.94 on the test set. Up-sampling of the minor class was quite effective as it allowed us to work with a balanced dataset without losing any information on the major class. This novel approach for HS prediction can now be freely applied by researchers through the SpotOn webserver.

SpotOn is an easy to use, publicly accessible web server that enables accurate identification of binding Hot-Spots in protein-protein complexes with minimal input requirements. The method at its back-end is robust and the most accurate to date as demonstrated here. A successful run will present the user with meaningful results displayed in user-friendly, interactive formats. It should be equally useful to experts in the field of computational structural biology as well as less computationally trained researchers. SpotOn is part of a family of widely-used web portals operated by the Utrecht group in the general area of biomolecular interaction. As such it is part of services for which we aim to provide both high reliability and availability.

## Methods

### Dataset Construction

We combined the ASEdb^15^, the BID^16^, PINT^17^ and SKEMPI^18^ databases to construct a non-redundant dataset of mutations. Collectively they provide experimental ΔΔG_binding_ values for interfacial residues for complexes for which there is an available three-dimensional (3D) structure in the Protein Databank^31^. To prevent repeated complexes, all sequences were filtered to ensure at most 35% sequence redundancy in each interface. Crystal structures were gathered from the Protein Data Bank^31^ and filtered so that only protein atoms were considered. Hydrogens were added by an in-house VMD^28^ script. A total of 534 mutations from 53 different complexes are comprised in our dataset.

### Sequence/Structural Features

Twelve solvent accessible surface area (SASA)-related features were calculated as described in previous works^8, 14^. Interfacial residue count was also added, totalling twenty features, each one corresponding to a single amino acid residue. Further as structural features, we calculated the intermolecular atomic contacts within 2.5 Å and 4.0 Å, and the number of intermolecular hydrophobic interactions. These were calculated using in-house VMD software^28^ scripts, which are incorporated in our pipeline.

Both PSSMs and the corresponding weighted observed percentages were computed using BLAST^29, 32^, providing forty additional features. PSSMs provide a relatively easy way of determining how likely is it to find a specific amino acid residue at a given position (positive scores indicate high likelihood, negative scores point towards low frequency). According to Lin *et al*.^33^, PSSM analysis can have shortcomings since the generation of PSSM of a given protein depends largely on the search dataset. Therefore, if not enough homologs are found during the BLAST search in PSSM, SpotON will return an error file to the user. We have extended the sequence related features to include those 805 extracted from the PROTR^34^ module from the R package: i) the Amino Acid Composition (ACC) of protein, the fraction of each amino acid type within the protein; ii) Pseudo Amino Acid Composition (PAAC)^35^ adds up to the standard 20 amino acid definition, providing information about patterns; iii) amphiphilic PAAC, a set of the twenty original amino acids, plus descriptors regarding the hydrophobicity/hydrophilicity of the sequences that have often displayed positive effects regarding protein-protein interaction prediction algorithms; iv) BLOcks Substitution Matrix (BLOSUM) which provides evolutionary features in the form of a scoring matrix upon sequence alignment taking into account amino acid substitution at a 62% level of similarity; v) Protein Fingerprinting, a process that allows for the identification and differentiation of proteins by unique characteristics, sometimes despite sequence similarity and is generated from both the AAindex and by PCA; vi) ProteoChemometric Modeling (PCM)^36^ derived from PCA of 2D and 3D descriptors, that provides a perspective regarding protein dynamics and interaction with ligands. We have to stress out that PAAC does not only include residues composition, but also long-range correlations of the physicochemical properties between two residues. It has been widely used in protein classification^37–41^. We therefore added it to our model to improve the final accuracy. We totalize a final of 881 features calculated for 534 observations, each one corresponding to an amino acid residue classified as HS or NS. From this, 55 are residue-based and the remaining are protein-based. We have written all the feature calculation code in Python and it is available upon request.

### Machine-Learning Techniques

Even though various software are available to perform machine-learning, we chose the R programming language^42^, together with the Classification And Regression Training (*caret*)^21^ package, allowing us to test several high quality machine-learning algorithms present in *caret* by using an intuitive and increasingly popular programming language. We randomly split our dataset into training and testing set, each consisting of 70% (374 mutations/observations) and 30% (160 mutations/observations) of the original dataset, respectively. In doing that we ensured that fraction of positive/negative cases is the same for all subsets of our original dataset. Accordingly, each of these sets contains equal proportions of HS and NS. Dealing with HS classification, requires dealing with unbalanced datasets, 127 HS versus 407 NS in the original dataset, which can have a negative impact on a model’s performance. Although, overcoming this problem can be done in several ways, we chose to perform both down-sampling and up-sampling. In the first, a random subset of all classes in the training is generated so that each class size matches the size of the least prevalent class. In up-sampling, random sampling of the minor class with replacement is performed so that the size of the minor class (HS) matches that of the major class (NS). The 51 algorithms tested were: Boruta, C5.0, C5.0Rules, C5.0Tree, LogitBoost, ORFlog, ORFpls, ORFridge, ORFsvm, RRF, RRFglobal, ada, adaboost, amdai, avNNet, bagEarth, bagEarthGCV, bagFDA, bagFDAGCV, ctree, ctree2, dwdPoly, dwdRadial, evtree, fda, gamboost, glm, glmboost, hdda, knn, lda, lda2, loclda, multinom, nb, pda, plr, qda, ranger, rda, rf, stepLDA, stepQDA, svmLinear, svmLinear2, svmPoly, svmRadial, svmRadialCost, svmRadialSigma, svmRadialWeights and wsrf.

N-fold cross-validation test, sub-sampling test, independent dataset test and jackknife cross-validation test have been widely used to examine the performance of a prediction model^38, 43–50^. In this study, all classification models were tested using 10-fold cross validation repeated 10 times in order to avoid overfitting and obtain the model’s generalization error. This means that the training set was split randomly into ten subsets, using nine of the them to train the model and taking the remaining one to test the final performance of the model. This process was repeated ten times. Two different sets were tested in which:

i) the variables were normalized;
ii) the variables were normalized and then subjected to PCA.

The validity and performance of the various methods was determined by measuring the Area Under the Receiver Operator Curve (AUROC), the Accuracy (eq. 1.1), True Positive Rate (TPR)/recall/sensitivity (eq. 1.2), True Negative Rate (TNR)/specificity, (eq. 1.3), Positive Predictive Value (PPV/Precision, eq. 1.4), Negative Predictive Value (NPV, eq. 1.5), False Discovery Rate (FDR, eq. 1.6), False Negative Rate (FNR, eq. 1.7), F1-score (eq. 1.8) and Mathew’s Correlation Coefficient (MCC, eq. 1.9).

1.1 $$Accuracy=\frac{TP+TN}{TP+FP+FN+TN}$$

1.2 $$TPR=\frac{TP}{TP+FN}$$

1.3 $$TNR=\frac{TN}{FP+TN}$$

1.4 $$PPV=\frac{TP}{TP+FP}$$

1.5 $$NPV=\frac{FP}{FN+TN}$$

1.6 $$FDR=\frac{FP}{FP+TP}=1-PPV$$

1.7 $$FNR=\frac{FN}{TP+FN}=1-TPR$$

1.8 $$F1-score=\frac{2TP}{2TP+FP+FN}$$

1.9 $$MCC=\frac{TP\times TN-FP\times FN}{\surd (TP+FP)(TP+FN)(TN+FP)(TN+FN)}\,$$

The equations determining the different metrics are calculated using four values: TP, TN, FP, FN. These stand for True Positive (the number of correctly classified HS), True Negative (the number of correctly classified NS), False Positive (the number of NS classified as HS) and False Negative (the number of HS classified as NS). The calculations for the various algorithms were performed with R.

To cluster the 51 used algorithms, the following Caret’s tags^21^ were used: Accepts Case Weights, Bagging, Bayesian Model, Binary Predictors Only, Boosting, Categorical Predictors Only, Cost Sensitive Learning, Discriminant Analysis, Distance Weighted Discrimination, Ensemble Model, Feature Extraction, Feature Extraction Models, Feature Selection Wrapper, Gaussian Process, Generalized Additive Model, Generalized Linear Model, Generalized Linear Models, Handle Missing Predictor Data, Implicit Feature Selection, Kernel Method, L1 Regularization, L1 Regularization Models, L2 Regularization, L2 Regularization Models, Linear Classifier, Linear Classifier Models, Linear Regression, Linear Regression Models, Logic Regression, Logistic Regression, Mixture Model, Model Tree, Multivariate Adaptive Regression Splines, Neural Network, Oblique Tree, Ordinal Outcomes, Partial Least Squares, Polynomial Model, Prototype Models, Quantile Regression, Radial Basis Function, Random Forest, Regularization, Relevance Vector Machines, Ridge Regression, Robust Methods, Robust Model, ROC Curves, Rule-Based Model, Self-Organizing Maps, String Kernel, Support Vector Machines, Text Mining, Tree-Based Model and Two Class Only. For all tags, a binary attribute was assigned with a value of 1 (if present) or 0 (if not present). The algorithms were subjected to hierarchical clustering which returned a distance matrix based on the Jaccard similarity coefficient and the complete aggregation scheme. The different clusters were compared by the parametric one way MANOVA to check if the groups differ from each other significantly in one or more characteristics. The two hypotheses tested are:

1.10 $${H}_{0}:{\mu }_{1}={\mu }_{2}=\ldots ={\mu }_{L}\,vs\,{H}_{1}:{\mu }_{r}\ne {\mu }_{s},for\,one\,pair\,r,s$$

MANOVA calculates the two matrices of between- and within-scatter:

1.11 $$H=k{\sum }_{l=1}^{L}(\overline{{x}_{l}}-\bar{x}){(\overline{{x}_{l}}-\bar{x})}^{T}$$

112 $$E=k{\sum }_{l=1}^{L}{\sum }_{j=1}^{K}({x}_{lj}-\overline{{x}_{l}}){({x}_{lj}-\overline{{x}_{l}})}^{T}$$

Considering that A = *H* × *E* ^−1^, four different statistics were calculated based on the eigenvalues *λ*_*p*_ of the A matrix: Pillai M S. Barlett trace

113 $${\lambda }_{Pillai}=tr({(I+A)}^{-1})$$

Logistic regression is used to model dichotomous outcome variables as in this logit (natural log of odds) model, the log odds of the outcome are modelled as a set of linear equations:

1.14 $${\rm{logit}}({\pi }_{i})=\,\mathrm{ln}(\frac{{\pi }_{i}}{1-{\pi }_{1}})=\sum _{i=1}^{n}{\beta }_{i}{X}_{i}$$

where *π*_*i*_ are the positive event occurrence probability, *βi* the element of the vector of regression coefficients and X_i_ the element of the vector of covariates. We have applied logit regression using as independent variables the binary classification attained by the top performer of each of the clusters attained above. We have also performed stepwise regression (bidirectional), a semi-automatic process of building a model by adding or removing variables based solely on the t-statistics of their estimated coefficients.

### Data availability

All data and features used to train SpotOn are available as supplementary material.

## Electronic supplementary material

Supplementary information

Supplementary Dataset 1
