# Supplementary material for: SpotOn: High Accuracy Identification of Protein-Protein Interface Hot-Spots
Source: Sci Rep. 2017 Aug 14;7:8007. doi: 10.1038/s41598-017-08321-2 (PMC5556074; doi:10.1038/s41598-017-08321-2)
Supplement: Supplementary file 1 — Supplementary information [file 41598_2017_8321_MOESM1_ESM.pdf]

SpotOn: High Accuracy Identification of Protein-Protein Interface Hot-Spots

Irina S. Moreira[1,2*#], Panagiotis I. Koukos[2#], Rita Melo[1,3], Jose G. Almeida[1], Antonio G. Preto[1], Joerg Schaarschmidt[2], Mikael Trellet[2], Zeynep H. Gümüş[4], Joaquim Costa[5], Alexandre M.J.J. Bonvin[2*]

[1] CNC - Center for Neuroscience and Cell Biology; Rua Larga, FMUC, Polo I, 1ºandar, Universidade de Coimbra, 3004-517, Coimbra, Portugal.

[2] Bijvoet Center for Biomolecular Research, Faculty of Science - Chemistry, Utrecht University, Utrecht, 3584CH, the Netherlands

[3] Centro de Ciências e Tecnologias Nucleares, Instituto Superior Técnico, Universidade de Lisboa, Estrada Nacional 10 (ao km 139,7), 2695-066 Bobadela LRS, Portugal

[4] Department of Genetics and Genomics and Icahn Institute for Genomics and Multiscale Biology, Icahn School of Medicine at Mount Sinai, New York, NY, USA

[5] CMUP/FCUP, Centro de Matemática da Universidade do Porto, Faculdade de Ciências, Rua do Campo Alegre, 4169-007 Porto, Portugal

# Joint first authors

*irina.moreira@cnc.uc.pt , a.m.j.j.bonvin@uu.nl

Table SI-1: Metrics for the training set upon PCA.

Clusters	Methods	ROC	Accuracy	Sensitivity	Specificity	PPV	NPV	FDR	FNR	F1-score	MCC
**Cluster I**	*avNNet*	0.80	0.97	0.93	0.98	0.94	0.98	0.06	0.07	0.94	0.91
	Boruta	0.78	1.00	1.00	1.00	1.00	1.00	0.00	0.00	1.00	1.00
	wsrf	0.77	1.00	0.99	1.00	1.00	1.00	0.00	0.00	0.99	0.99
	RRF	0.67	1.00	1.00	1.00	1.00	1.00	0.00	0.00	1.00	1.00
	RRFglobal	0.78	1.00	1.00	1.00	1.00	1.00	0.00	0.00	1.00	1.00
	ranger	0.22	0.01	0.02	0.00	0.01	0.00	0.99	1.00	0.01	-0.99
	rf	0.78	1.00	1.00	1.00	1.00	1.00	0.00	0.00	1.00	1.00
**Cluster II**	*ORFridge*	0.80	1.00	0.99	1.00	1.00	1.00	0.00	0.01	0.99	0.99
	ORFsvm	0.80	0.99	0.98	1.00	0.99	0.99	0.01	0.02	0.98	0.99
	ORFlog	0.80	1.00	0.99	1.00	1.00	1.00	0.00	0.01	0.99	0.99
	ORFpls	0.79	0.99	0.98	1.00	1.00	0.99	0.00	0.02	0.99	0.99
	LogitBoost	0.73	0.93	0.78	0.97	0.90	0.93	0.10	0.03	0.83	0.79
	ada	0.79	0.94	0.78	0.99	0.95	0.93	0.05	0.22	0.85	0.83
	adaboost	0.51	1.00	1.00	1.00	1.00	1.00	0.00	0.00	1.00	1.00
	gamboost	0.76	0.82	0.34	0.98	0.81	0.82	0.19	0.66	0.48	0.46
	evtree	0.52	0.85	0.54	0.95	0.77	0.87	0.23	0.05	0.64	0.56
	ctree	0.65	0.84	0.61	0.92	0.70	0.88	0.30	0.39	0.65	0.56
	ctree2	0.55	0.80	0.43	0.92	0.63	0.84	0.37	0.57	0.51	0.40
	C5.0	0.77	1.00	1.00	1.00	1.00	1.00	0.00	0.00	1.00	1.00
	C5.0Rules	0.56	0.91	0.67	0.99	0.95	0.91	0.05	0.33	0.79	0.75
	C5.0Tree	0.63	0.94	0.74	1.00	1.00	0.93	0.00	0.26	0.85	0.83
	fda	0.71	0.82	0.44	0.94	0.71	0.84	0.29	0.56	0.54	0.45
	bagFDAGCV	0.77	0.86	0.51	0.97	0.83	0.86	0.17	0.03	0.63	0.58
	bagFDA	0.77	0.85	0.48	0.97	0.83	0.86	0.17	0.52	0.61	0.56
	bagEarth	0.78	0.87	0.54	0.97	0.84	0.87	0.16	0.46	0.66	0.61
	bagEarthGCV	0.78	0.87	0.55	0.98	0.88	0.87	0.13	0.02	0.68	0.64
**Cluster III**	*glmboost*	0.75	0.81	0.24	0.99	0.84	0.81	0.16	0.76	0.37	0.40
	glm	0.72	0.86	0.62	0.93	0.74	0.89	0.26	0.38	0.67	0.58
	multinom	0.73	0.86	0.61	0.94	0.76	0.88	0.24	0.39	0.68	0.60
	plr	0.73	0.86	0.61	0.94	0.75	0.88	0.25	0.39	0.67	0.60
**Cluster IV**	*svmRadialCost*	0.81	0.83	0.31	0.99	0.87	0.82	0.13	0.69	0.46	0.47
	svmRadial	0.80	0.83	0.36	0.98	0.82	0.83	0.18	0.64	0.50	0.48
	svmRadialSigma	0.79	0.82	0.34	0.98	0.81	0.82	0.19	0.02	0.48	0.46
	svmPoly	0.79	0.81	0.25	0.99	0.85	0.81	0.15	0.75	0.38	0.41
	svmLinear	0.76	0.78	0.07	1.00	1.00	0.77	0.00	0.00	0.13	0.23
	svmLinear2	0.76	0.85	0.51	0.95	0.78	0.86	0.22	0.05	0.61	0.54
	svmRadialWeights	0.77	0.80	0.21	0.99	0.86	0.80	0.14	0.01	0.34	0.37
	dwdPoly	0.72	0.83	0.38	0.97	0.79	0.83	0.21	0.03	0.52	0.47
	dwdRadial	0.73	1.00	1.00	1.00	1.00	1.00	0.00	0.00	1.00	1.00
**Cluster V**	*rda*	0.77	0.83	0.51	0.93	0.70	0.86	0.30	0.49	0.59	0.49
	stepLDA	0.61	0.77	0.06	0.99	0.71	0.77	0.29	0.94	0.10	0.15
	loclda	0.75	0.93	0.79	0.97	0.90	0.94	0.10	0.03	0.84	0.79
	lda2	0.76	0.83	0.51	0.93	0.70	0.86	0.30	0.49	0.59	0.49
	hdda	0.72	0.83	0.47	0.94	0.72	0.85	0.28	0.06	0.57	0.48
	lda	0.76	0.83	0.51	0.93	0.70	0.86	0.30	0.49	0.59	0.49
	amdai	0.76	0.83	0.51	0.93	0.70	0.86	0.30	0.07	0.59	0.49
	stepQDA	0.63	0.78	0.15	0.98	0.65	0.79	0.35	0.85	0.24	0.25
	pda	0.76	0.83	0.51	0.93	0.70	0.86	0.30	0.07	0.59	0.49
	qda	0.64	0.76	0.91	0.71	0.50	0.96	0.50	0.09	0.64	0.53
	knn	0.69	0.80	0.26	0.97	0.74	0.81	0.26	0.74	0.38	0.35
	nb	0.72	0.84	0.64	0.90	0.67	0.89	0.33	0.36	0.66	0.55

Table SI-2: Metrics for the training set upon PCA and up-sampling.

Clusters	Methods	ROC	Accuracy	Sensitivity	Specificity	PPV	NPV	FDR	FNR	F1-score	MCC
**Cluster I**	*avNNet*	0.94	0.96	0.97	0.95	0.96	0.97	0.04	0.03	0.96	0.88
	Boruta	0.98	1.00	1.00	1.00	1.00	1.00	0.00	0.00	1.00	1.00
	wsrf	0.98	1.00	1.00	1.00	1.00	1.00	0.00	0.00	1.00	1.00
	RRF	0.95	1.00	1.00	1.00	1.00	1.00	0.00	0.00	1.00	1.00
	RRFglobal	0.99	1.00	1.00	1.00	1.00	1.00	0.00	0.00	1.00	1.00
	ranger	0.98	1.00	1.00	1.00	1.00	1.00	0.00	0.00	1.00	1.00
	rf	0.98	1.00	1.00	1.00	1.00	1.00	0.00	0.00	1.00	1.00
**Cluster II**	*ORFridge*	0.98	1.00	1.00	1.00	1.00	1.00	0.00	0.00	1.00	1.00
	ORFsvm	0.98	1.00	1.00	1.00	1.00	1.00	0.00	0.00	1.00	1.00
	ORFlog	0.99	1.00	1.00	1.00	1.00	1.00	0.00	0.00	1.00	1.00
	ORFpls	0.98	1.00	1.00	1.00	1.00	1.00	0.00	0.00	1.00	1.00
	LogitBoost	0.88	0.92	0.95	0.89	0.9	0.94	0.1	0.11	0.92	0.77
	ada	0.95	0.98	0.99	0.97	0.97	0.99	0.03	0.01	0.98	0.93
	adaboost	0.79	1.00	1.00	1.00	1.00	1.00	0.00	0.00	1.00	1.00
	gamboost	0.83	0.82	0.83	0.81	0.81	0.83	0.19	0.17	0.82	0.57
	evtree	0.76	0.89	0.93	0.85	0.86	0.92	0.14	0.15	0.89	0.70
	ctree	0.87	0.92	0.93	0.91	0.91	0.92	0.09	0.07	0.92	0.79
	ctree2	0.67	0.68	0.49	0.87	0.79	0.63	0.21	0.51	0.61	0.37
	C5.0	0.98	1.00	1.00	1.00	1.00	1.00	0.00	0.00	1.00	1.00
	C5.0Rules	0.86	0.96	0.98	0.94	0.95	0.97	0.05	0.02	0.96	0.87
	C5.0Tree	0.88	0.99	0.99	0.99	0.99	0.99	0.01	0.01	0.99	0.97
	fda	0.81	0.80	0.83	0.77	0.78	0.82	0.22	0.17	0.81	0.53
	bagFDAGCV	0.86	0.84	0.86	0.81	0.82	0.85	0.18	0.19	0.84	0.60
	bagFDA	0.85	0.84	0.87	0.81	0.82	0.86	0.18	0.13	0.84	0.61
	bagEarth	0.86	0.84	0.85	0.82	0.83	0.84	0.17	0.15	0.84	0.60
	bagEarthGCV	0.86	0.85	0.86	0.83	0.84	0.86	0.16	0.17	0.85	0.62
**Cluster III**	*glmboost*	0.81	0.79	0.81	0.76	0.78	0.80	0.22	0.19	0.79	0.50
	glm	0.83	0.86	0.87	0.85	0.85	0.87	0.15	0.13	0.86	0.66
	multinom	0.88	0.86	0.88	0.85	0.85	0.88	0.15	0.12	0.87	0.66
	plr	0.88	0.86	0.88	0.85	0.85	0.88	0.15	0.12	0.87	0.66
**Cluster IV**	*svmRadialCost*	0.85	0.83	0.87	0.78	0.80	0.86	0.20	0.13	0.83	0.57
	svmRadial	0.87	0.83	0.87	0.79	0.81	0.86	0.19	0.13	0.84	0.58
	svmRadialSigma	0.87	0.84	0.87	0.80	0.81	0.86	0.19	0.20	0.84	0.59
	svmPoly	0.95	0.99	0.99	0.99	0.99	0.99	0.01	0.01	0.99	0.97
	svmLinear	0.88	0.87	0.94	0.80	0.82	0.93	0.18	0.20	0.88	0.65
	svmLinear2	0.88	0.88	0.93	0.83	0.84	0.92	0.16	0.17	0.88	0.68
	svmRadialWeights	0.91	0.80	0.97	0.64	0.73	0.95	0.27	0.36	0.83	0.52
	dwdRadial	0.80	1.00	1.00	1.00	1.00	1.00	0.00	0.00	1.00	1.00
**Cluster V**	*rda*	0.85	0.82	0.86	0.79	0.80	0.85	0.20	0.14	0.83	0.58
	stepLDA	0.61	0.61	0.69	0.53	0.60	0.63	0.40	0.31	0.64	0.19
	lda2	0.85	0.82	0.86	0.79	0.80	0.85	0.20	0.14	0.83	0.58
	hdda	0.76	0.81	0.78	0.85	0.84	0.79	0.16	0.15	0.81	0.59
	lda	0.85	0.82	0.86	0.79	0.80	0.85	0.20	0.14	0.83	0.58
	amdai	0.85	0.82	0.86	0.79	0.80	0.85	0.20	0.21	0.83	0.58
	stepQDA	0.63	0.62	0.78	0.46	0.59	0.68	0.41	0.22	0.67	0.21
	pda	0.85	0.82	0.86	0.79	0.80	0.85	0.20	0.21	0.83	0.58
	qda	0.82	0.83	0.90	0.77	0.80	0.88	0.20	0.10	0.84	0.59
	knn	0.85	0.84	0.95	0.72	0.77	0.94	0.23	0.05	0.85	0.57
	nb	0.83	0.82	0.81	0.82	0.82	0.81	0.18	0.19	0.81	0.57

Table SI-3: Metrics for the training set upon PCA and down-sampling.

Clusters	Methods	ROC	Accuracy	Sensitivity	Specificity	PPV	NPV	FDR	FNR	F1-score	MCC
**Cluster I**	*avNNet*	0.82	0.97	0.97	0.97	0.97	0.97	0.03	0.03	0.97	0.92
	Boruta	0.17	1.00	1.00	1.00	1.00	1.00	0.00	0.00	1.00	1.00
	wsrf	0.75	1.00	1.00	1.00	1.00	1.00	0.00	0.00	1.00	1.00
	RRF	0.64	1.00	1.00	1.00	1.00	1.00	0.00	0.00	1.00	1.00
	RRFglobal	0.77	1.00	1.00	1.00	1.00	1.00	0.00	0.00	1.00	1.00
	ranger	0.76	0.99	0.99	1.00	1.00	0.99	0.00	0.00	0.99	0.99
	rf	0.77	1.00	1.00	1.00	1.00	1.00	0.00	0.00	1.00	1.00
**Cluster II**	*ORFridge*	0.80	0.99	0.99	1.00	1.00	0.99	0.00	0.01	0.99	0.99
	ORFsvm	0.80	0.99	0.99	1.00	1.00	0.99	0.00	0.01	0.99	0.99
	ORFlog	0.80	1.00	1.00	1.00	1.00	1.00	0.00	0.00	1.00	1.00
	ORFpls	0.79	1.00	1.00	1.00	1.00	1.00	0.00	0.00	1.00	1.00
	LogitBoost	0.70	0.90	0.91	0.90	0.90	0.91	0.10	0.10	0.91	0.76
	ada	0.81	0.99	0.99	0.99	0.99	0.99	0.01	0.01	0.99	0.97
	adaboost	0.59	1.00	1.00	1.00	1.00	1.00	0.00	0.00	1.00	1.00
	gamboost	0.73	0.85	0.81	0.89	0.88	0.82	0.12	0.19	0.84	0.67
	evtree	0.63	0.85	0.87	0.83	0.84	0.86	0.16	0.17	0.85	0.63
	ctree	0.68	0.85	0.91	0.79	0.81	0.90	0.19	0.09	0.86	0.62
	ctree2	0.64	0.77	0.92	0.62	0.71	0.89	0.29	0.08	0.80	0.46
	C5.0	0.79	1.00	1.00	1.00	1.00	1.00	0.00	0.00	1.00	1.00
	C5.0Rules	0.63	0.94	0.92	0.96	0.95	0.92	0.05	0.08	0.94	0.87
	C5.0Tree	0.67	0.96	0.93	0.99	0.99	0.94	0.01	0.07	0.96	0.93
	fda	0.71	0.85	0.85	0.84	0.84	0.85	0.16	0.15	0.85	0.63
	bagFDAGCV	0.77	0.89	0.88	0.90	0.90	0.88	0.10	0.10	0.89	0.74
	bagFDA	0.74	0.88	0.84	0.92	0.91	0.85	0.09	0.16	0.88	0.74
	bagEarth	0.76	0.91	0.90	0.92	0.92	0.90	0.08	0.10	0.91	0.78
	bagEarthGCV	0.76	0.92	0.92	0.91	0.91	0.92	0.09	0.09	0.92	0.78
**Cluster III**	*glmboost*	0.75	0.83	0.84	0.82	0.82	0.84	0.18	0.16	0.83	0.59
	glm	0.71	0.88	0.88	0.89	0.89	0.88	0.11	0.12	0.88	0.72
	multinom	0.73	0.87	0.87	0.88	0.88	0.87	0.12	0.13	0.87	0.70
	plr	0.74	0.88	0.88	0.88	0.88	0.88	0.12	0.12	0.88	0.71
**Cluster IV**	*svmRadialCost*	0.78	0.82	0.80	0.84	0.84	0.81	0.16	0.20	0.82	0.59
	svmRadial	0.78	0.83	0.80	0.85	0.85	0.81	0.15	0.20	0.82	0.60
	svmRadialSigma	0.76	0.78	0.80	0.76	0.77	0.79	0.23	0.24	0.78	0.49
	svmPoly	0.79	0.9	0.89	0.92	0.92	0.89	0.08	0.11	0.90	0.77
	svmLinear	0.75	0.87	0.87	0.88	0.88	0.87	0.13	0.12	0.87	0.70
	svmLinear2	0.77	0.88	0.89	0.87	0.87	0.89	0.13	0.13	0.88	0.70
	svmRadialWeights	0.72	0.8	0.75	0.85	0.84	0.78	0.16	0.15	0.79	0.56
**Cluster V**	*rda*	0.78	0.86	0.83	0.89	0.88	0.84	0.12	0.17	0.86	0.68
	stepLDA	0.59	0.6	0.73	0.47	0.58	0.64	0.42	0.27	0.65	0.17
	lda2	0.77	0.86	0.83	0.89	0.88	0.84	0.12	0.17	0.86	0.68
	hdda	0.74	0.78	0.78	0.79	0.78	0.78	0.22	0.21	0.78	0.51
	lda	0.77	0.86	0.83	0.89	0.88	0.84	0.12	0.17	0.86	0.68
	amdai	0.73	0.84	0.80	0.88	0.87	0.81	0.13	0.12	0.83	0.64
	stepQDA	0.61	0.61	0.45	0.78	0.67	0.58	0.33	0.55	0.54	0.22
	pda	0.74	0.84	0.80	0.88	0.87	0.81	0.13	0.12	0.83	0.64
	qda	0.68	0.84	0.69	0.99	0.98	0.76	0.02	0.31	0.81	0.77
	knn	0.70	0.81	0.79	0.84	0.83	0.80	0.17	0.21	0.81	0.58
	nb	0.71	0.81	0.72	0.90	0.88	0.76	0.12	0.28	0.79	0.61

Table SI-4: Metrics for the training set upon z-scoring.

Clusters	Methods	ROC	Accuracy	Sensitivity	Specificity	PPV	NPV	FDR	FNR	F1-score	MCC
**Cluster I**	*avNNet*	0.77	0.93	0.90	0.94	0.83	0.97	0.17	0.10	0.86	0.82
	wsrf	0.77	0.99	0.99	0.99	0.98	1.00	0.02	0.01	0.98	0.97
	RRF	0.72	1.00	1.00	1.00	1.00	1.00	0.00	0.00	1.00	1.00
	RRFglobal	0.83	1.00	1.00	1.00	1.00	1.00	0.00	0.00	1.00	1.00
	ranger	0.17	0.14	0.48	0.03	0.13	0.16	0.87	0.97	0.21	-0.59
	rf	0.83	1.00	1.00	1.00	1.00	1.00	0.00	0.00	1.00	1.00
**Cluster II**	*bagEarth*	0.83	0.89	0.69	0.95	0.81	0.91	0.19	0.31	0.74	0.68
	bagEarthGCV	0.83	0.90	0.71	0.96	0.86	0.91	0.14	0.04	0.78	0.71
	bagFDAGCV	0.83	0.89	0.66	0.95	0.82	0.90	0.18	0.05	0.73	0.66
	C5.0	0.81	1.00	1.00	1.00	1.00	1.00	0.00	0.00	1.00	1.00
	C5.0Rules	0.64	0.96	0.84	0.99	0.97	0.95	0.03	0.16	0.90	0.87
	C5.0Tree	0.76	0.98	0.94	0.99	0.98	0.98	0.02	0.06	0.96	0.94
	ctree	0.67	0.84	0.79	0.86	0.63	0.93	0.37	0.21	0.70	0.61
	ctree2	0.66	0.82	0.63	0.88	0.63	0.88	0.37	0.37	0.63	0.51
	evtree	0.55	0.87	0.54	0.97	0.86	0.87	0.14	0.03	0.66	0.61
	fda	0.80	0.83	0.53	0.93	0.70	0.86	0.30	0.47	0.60	0.51
	LogitBoost	0.75	0.88	0.64	0.95	0.80	0.89	0.20	0.05	0.71	0.64
	ORFlog	0.74	0.87	0.70	0.93	0.75	0.91	0.25	0.30	0.72	0.65
	ORFpls	0.75	0.97	0.87	1.00	1.00	0.96	0.00	0.13	0.93	0.91
	ORFridge	0.75	0.97	0.89	1.00	1.00	0.97	0.00	0.11	0.94	0.93
	ORFsvm	0.80	0.99	0.97	1.00	1.00	0.99	0.00	0.03	0.98	0.98
**Cluster III**	*glmboost*	0.81	0.82	0.34	0.97	0.79	0.82	0.21	0.66	0.47	0.43
	glm	0.56	0.91	0.78	0.95	0.83	0.93	0.17	0.22	0.80	0.75
	multinom	0.73	0.93	0.82	0.96	0.88	0.95	0.12	0.18	0.85	0.79
	plr	0.71	0.93	0.83	0.96	0.88	0.95	0.12	0.17	0.86	0.80
**Cluster IV**	*dwdPoly*	0.73	0.85	0.45	0.97	0.83	0.85	0.17	0.03	0.58	0.53
	dwdRadial	0.70	1.00	0.99	1.00	1.00	1.00	0.00	0.00	0.99	0.99
	svmLinear	0.75	0.83	0.28	1.00	1.00	0.82	0.00	0.00	0.44	0.48
	svmLinear2	0.74	0.93	0.79	0.97	0.89	0.94	0.11	0.03	0.83	0.79
	svmPoly	0.79	0.89	0.58	0.98	0.90	0.88	0.10	0.42	0.71	0.66
	svmRadial	0.78	0.82	0.31	0.98	0.85	0.82	0.15	0.69	0.46	0.43
	svmRadialCost	0.78	0.81	0.22	0.99	0.87	0.80	0.13	0.78	0.36	0.38
	svmRadialSigma	0.78	0.80	0.17	1.00	0.94	0.79	0.06	0.00	0.29	0.37
	svmRadialWeights	0.74	0.76	0.01	1.00	1.00	0.76	0.00	0.00	0.02	0.09
**Cluster V**	*hdda*	0.71	0.81	0.42	0.94	0.67	0.84	0.33	0.06	0.51	0.43
	knn	0.67	0.8	0.27	0.97	0.73	0.81	0.27	0.73	0.39	0.36
	lda	0.71	0.91	0.74	0.96	0.85	0.92	0.15	0.26	0.79	0.74
	lda2	0.71	0.91	0.74	0.96	0.85	0.92	0.15	0.26	0.79	0.74
	nb	0.50	0.81	0.67	0.85	0.59	0.89	0.41	0.33	0.63	0.50
	pda	0.71	0.9	0.74	0.95	0.84	0.92	0.16	0.05	0.79	0.72
	stepLDA	0.67	0.78	0.25	0.94	0.56	0.80	0.44	0.06	0.34	0.26
	stepQDA	0.60	0.78	0.11	0.99	0.77	0.78	0.23	0.89	0.20	0.24

Table SI-5: Metrics for the training set upon z-scoring and up-sampling.

Clusters	Methods	ROC	Accuracy	Sensitivity	Specificity	PPV	NPV	FDR	FNR	F1-score	MCC
**Cluster I**	*avNNet*	0.90	0.95	0.96	0.94	0.94	0.96	0.06	0.04	0.95	0.86
	wsrf	0.98	1.00	1.00	1.00	1.00	1.00	0.00	0.00	1.00	1.00
	RRF	0.97	1.00	1.00	1.00	1.00	1.00	0.00	0.00	1.00	1.00
	RRFglobal	0.98	1.00	1.00	1.00	1.00	1.00	0.00	0.00	1.00	1.00
	ranger	0.97	1.00	1.00	1.00	1.00	1.00	0.00	0.00	1.00	1.00
	rf	0.98	1.00	1.00	1.00	1.00	1.00	0.00	0.00	1.00	1.00
**Cluster II**	*ORFridge*	0.96	1.00	1.00	1.00	1.00	1.00	0.00	0.00	1.00	1.00
	ORFsvm	0.98	1.00	1.00	1.00	1.00	1.00	0.00	0.00	1.00	1.00
	ORFlog	0.9	0.92	0.92	0.92	0.92	0.92	0.08	0.08	0.92	0.80
	ORFpls	0.96	0.99	0.98	1.00	1.00	0.98	0.00	0.02	0.99	0.99
	LogitBoost	0.89	0.93	0.95	0.91	0.91	0.95	0.09	0.09	0.93	0.80
	evtree	0.78	0.88	0.94	0.83	0.85	0.93	0.15	0.17	0.89	0.69
	ctree	0.88	0.91	0.94	0.88	0.89	0.93	0.11	0.06	0.91	0.75
	ctree2	0.81	0.80	0.88	0.73	0.76	0.86	0.24	0.12	0.82	0.53
	C5.0	0.98	1.00	1.00	1.00	1.00	1.00	0.00	0.00	1.00	1.00
	C5.0Rules	0.87	0.97	0.98	0.96	0.97	0.98	0.03	0.02	0.97	0.91
	C5.0Tree	0.89	0.99	1.00	0.98	0.98	1.00	0.02	0.00	0.99	0.96
	fda	0.89	0.87	0.91	0.83	0.84	0.9	0.16	0.09	0.88	0.66
	bagFDAGCV	0.91	0.90	0.96	0.85	0.86	0.95	0.14	0.15	0.91	0.73
	bagEarth	0.91	0.91	0.93	0.89	0.90	0.93	0.10	0.07	0.91	0.76
	bagEarthGCV	0.91	0.90	0.91	0.89	0.89	0.91	0.11	0.11	0.90	0.74
**Cluster III**	*glmboost*	0.87	0.85	0.91	0.79	0.81	0.9	0.19	0.09	0.86	0.62
	glm	0.76	0.87	0.85	0.88	0.88	0.86	0.12	0.15	0.87	0.68
	multinom	0.87	0.94	0.97	0.92	0.92	0.97	0.08	0.03	0.94	0.83
	plr	0.87	0.94	0.96	0.92	0.92	0.96	0.08	0.08	0.94	0.83
**Cluster IV**	*svmRadialCost*	0.84	0.75	0.80	0.71	0.73	0.78	0.27	0.2	0.76	0.44
	svmRadial	0.85	0.79	0.81	0.76	0.77	0.80	0.23	0.19	0.79	0.50
	svmRadialSigma	0.85	0.78	0.81	0.75	0.77	0.80	0.23	0.25	0.79	0.49
	svmPoly	0.98	1.00	1.00	1.00	1.00	1.00	0.00	0.00	1.00	1.00
	svmLinear	0.87	0.94	0.97	0.91	0.92	0.97	0.08	0.09	0.94	0.82
	svmLinear2	0.87	0.93	0.95	0.91	0.91	0.95	0.09	0.09	0.93	0.80
	svmRadialWeights	0.91	0.74	0.97	0.52	0.67	0.94	0.33	0.48	0.79	0.43
	dwdRadial	0.75	1.00	1.00	1.00	1.00	1.00	0.00	0.00	1.00	1.00
**Cluster V**	*stepLDA*	0.74	0.73	0.82	0.64	0.70	0.78	0.30	0.18	0.75	0.39
	lda2	0.87	0.91	0.94	0.88	0.88	0.93	0.12	0.06	0.91	0.75
	hdda	0.76	0.91	0.88	0.95	0.95	0.89	0.05	0.05	0.91	0.82
	lda	0.87	0.91	0.94	0.88	0.88	0.93	0.12	0.06	0.91	0.75
	stepQDA	0.71	0.68	0.89	0.46	0.62	0.81	0.38	0.54	0.73	0.31
	pda	0.87	0.91	0.94	0.88	0.89	0.93	0.11	0.12	0.91	0.75
	knn	0.83	0.84	0.95	0.72	0.77	0.94	0.23	0.05	0.85	0.57
	nb	0.75	0.78	0.84	0.72	0.75	0.82	0.25	0.16	0.79	0.48

Table SI-6: Metrics for the training set upon z-scoring and down-sampling.

Clusters	Methods	ROC	Accuracy	Sensitivity	Specificity	PPV	NPV	FDR	FNR	F1-score	MCC
**Cluster I**	*avNNet*	0.79	0.98	0.99	0.97	0.97	0.99	0.03	0.01	0.98	0.93
	wsrf	0.82	0.99	0.99	0.99	0.99	0.99	0.01	0.01	0.99	0.97
	RRF	0.82	1.00	1.00	1.00	1.00	1.00	0.00	0.00	1.00	1.00
	RRFglobal	0.85	1.00	1.00	1.00	1.00	1.00	0.00	0.00	1.00	1.00
	ranger	0.84	0.99	0.99	1.00	1.00	0.99	0.00	0.00	0.99	0.99
	rf	0.84	1.00	1.00	1.00	1.00	1.00	0.00	0.00	1.00	1.00
**Cluster II**	*ORFridge*	0.76	0.99	0.99	0.99	0.99	0.99	0.01	0.01	0.99	0.97
	ORFsvm	0.79	0.99	0.99	0.99	0.99	0.99	0.01	0.01	0.99	0.97
	ORFlog	0.73	0.85	0.75	0.96	0.94	0.79	0.06	0.25	0.84	0.74
	ORFpls	0.76	0.99	0.99	0.99	0.99	0.99	0.01	0.01	0.99	0.97
	LogitBoost	0.78	0.90	0.85	0.96	0.95	0.87	0.05	0.04	0.90	0.82
	evtree	0.75	0.82	0.89	0.75	0.78	0.87	0.22	0.25	0.83	0.56
	ctree	0.69	0.77	0.70	0.84	0.82	0.74	0.18	0.30	0.75	0.51
	ctree2	0.70	0.80	0.92	0.69	0.75	0.90	0.25	0.08	0.82	0.52
	C5.0	0.83	1.00	1.00	1.00	1.00	1.00	0.00	0.00	1.00	1.00
	C5.0Rules	0.74	0.92	0.88	0.97	0.96	0.89	0.04	0.12	0.92	0.86
	C5.0Tree	0.74	0.97	0.96	0.99	0.99	0.96	0.01	0.04	0.97	0.95
	fda	0.85	0.88	0.89	0.87	0.87	0.89	0.13	0.11	0.88	0.70
	bagFDAGCV	0.85	0.94	0.94	0.93	0.93	0.94	0.07	0.07	0.94	0.83
	bagEarth	0.86	0.98	0.99	0.98	0.98	0.99	0.02	0.01	0.98	0.95
	bagEarthGCV	0.86	0.97	0.98	0.97	0.97	0.98	0.03	0.03	0.97	0.93
**Cluster III**	*glmboost*	0.82	0.88	0.87	0.89	0.89	0.87	0.11	0.13	0.88	0.71
	glm	0.59	1.00	1.00	1.00	1.00	1.00	0.00	0.00	1.00	1.00
	multinom	0.69	0.97	0.97	0.97	0.97	0.97	0.03	0.03	0.97	0.92
	plr	0.69	0.97	0.97	0.97	0.97	0.97	0.03	0.03	0.97	0.92
**Cluster IV**	*svmRadialCost*	0.75	0.78	0.72	0.83	0.81	0.75	0.19	0.28	0.76	0.51
	svmRadial	0.74	0.79	0.74	0.83	0.81	0.76	0.19	0.26	0.78	0.53
	svmRadialSigma	0.73	0.78	0.72	0.84	0.82	0.75	0.18	0.16	0.77	0.52
	svmPoly	0.79	0.89	0.91	0.88	0.88	0.91	0.12	0.09	0.90	0.73
	svmLinear	0.70	0.97	0.98	0.97	0.97	0.98	0.03	0.03	0.97	0.93
	svmLinear2	0.70	0.95	0.96	0.94	0.94	0.95	0.06	0.06	0.95	0.86
	svmRadialWeights	0.70	0.79	0.72	0.85	0.83	0.75	0.17	0.15	0.77	0.54
**Cluster V**	*stepLDA*	0.75	0.73	0.58	0.88	0.83	0.68	0.17	0.12	0.68	0.47
	lda2	0.65	0.92	0.91	0.92	0.92	0.91	0.08	0.09	0.92	0.79
	hdda	0.72	0.81	0.74	0.88	0.86	0.77	0.14	0.12	0.80	0.60
	lda	0.65	0.92	0.91	0.92	0.92	0.91	0.08	0.09	0.92	0.79
	stepQDA	0.71	0.78	0.78	0.79	0.78	0.78	0.22	0.21	0.78	0.51
	pda	0.65	0.95	0.94	0.96	0.95	0.94	0.05	0.04	0.95	0.88
	knn	0.68	0.72	0.66	0.78	0.75	0.70	0.25	0.34	0.70	0.40
	nb	0.50	0.80	0.72	0.89	0.86	0.76	0.14	0.28	0.79	0.60

Table SI-7: P-values for the one-way MANOVA performed on the means scores for the sensitivity, specificity and AUROC of all pre-processing conditions.

PRE-PROCESSING	MANOVA P-VALUE
PCA	0.003
PCAUP	0.001
PCADOWN	0.000
SCALED	0.004
SCALEDUP	0.025
SCALEDDOWN	0.004

Table SI-8: Metrics for the best methods of each cluster for all pre-processing conditions and their respective means, as calculated for the training set.

	Cluster I	Cluster II	Cluster III	Cluster IV	Cluster V	Mean Values
**PCA**	*avNNet*	*ORFlog*	*glmboost*	*svmRadialCost*	*rda*	**PCA**
**AUROC**	0.80	0.80	0.75	0.81	0.77	0.79
**Accuracy**	0.97	1.00	0.81	0.83	0.83	0.89
**Sensitivity**	0.93	0.99	0.24	0.31	0.51	0.60
**Specificity**	0.98	1.00	0.99	0.99	0.93	0.98
**PPV**	0.94	1.00	0.84	0.87	0.70	0.87
**NPV**	0.98	1.00	0.81	0.82	0.86	0.89
**FPR**	0.06	0.00	0.16	0.13	0.30	0.13
**F1-score**	0.94	0.99	0.37	0.46	0.59	0.67
**MCC**	0.93	0.99	0.40	0.47	0.49	0.66
**PCAUp**	*RRFglobal*	*ORFlog*	*Plr*	*svmPoly*	*amdai*	**PCAUp**
**AUROC**	0.99	0.99	0.88	0.95	0.85	0.93
**Accuracy**	1.00	1.00	0.86	0.99	0.82	0.93
**Sensitivity**	1.00	1.00	0.88	0.99	0.86	0.95
**Specificity**	1.00	1.00	0.85	0.99	0.79	0.93
**PPV**	1.00	1.00	0.85	0.99	0.80	0.93
**NPV**	1.00	1.00	0.88	0.99	0.85	0.94
**FPR**	0.00	0.00	0.15	0.01	0.20	0.07
**F1-score**	1.00	1.00	0.87	0.99	0.83	0.94
**MCC**	1.00	1.00	0.66	0.97	0.58	0.84
**PCADown**	*avNNet*	*ada*	*glmboost*	*svmPoly*	*Rda*	**PCADown**
**AUROC**	0.82	0.81	0.75	0.79	0.78	0.79
**Accuracy**	0.97	0.99	0.83	0.90	0.86	0.91
**Sensitivity**	0.97	0.99	0.84	0.89	0.83	0.90
**Specificity**	0.97	0.99	0.82	0.92	0.89	0.92
**PPV**	0.97	0.99	0.82	0.92	0.88	0.92
**NPV**	0.97	0.99	0.84	0.89	0.84	0.91
**FPR**	0.03	0.01	0.18	0.08	0.12	0.08
**F1-score**	0.97	0.99	0.83	0.90	0.86	0.91
**MCC**	0.92	0.97	0.59	0.77	0.68	0.79
**Scaled**	*RRFglobal*	*bagEarthGCV*	*glmboost*	*svmPoly*	*Pda*	**Scaled**
**AUROC**	0.83	0.83	0.81	0.79	0.71	0.79
**Accuracy**	1.00	0.90	0.82	0.89	0.90	0.90
**Sensitivity**	1.00	0.71	0.34	0.58	0.74	0.67
**Specificity**	1.00	0.96	0.97	0.98	0.95	0.97
**PPV**	1.00	0.86	0.79	0.90	0.84	0.88
**NPV**	1.00	0.91	0.82	0.88	0.92	0.91
**FPR**	0.00	0.14	0.21	0.10	0.16	0.12
**F1-score**	1.00	0.78	0.47	0.71	0.79	0.75
**MCC**	1.00	0.71	0.43	0.66	0.72	0.70
**ScaledUp**	*rf*	*ORFsvm*	*plr*	*svmPoly*	*pda*	**ScaledUp**
**AUROC**	0.98	0.98	0.87	0.98	0.87	0.94
**Accuracy**	1.00	1.00	0.94	1.00	0.91	0.97
**Sensitivity**	1.00	1.00	0.96	1.00	0.94	0.98

**Specificity**	1.00	1.00	0.92	1.00	0.88	0.96
**PPV**	1.00	1.00	0.92	1.00	0.89	0.96
**NPV**	1.00	1.00	0.96	1.00	0.93	0.98
**FPR**	0.00	0.00	0.08	0.00	0.11	0.04
**F1-score**	1.00	1.00	0.94	1.00	0.91	0.97
**MCC**	1.00	1.00	0.83	1.00	0.75	0.92
**ScaledDown**	*RRFglobal*	*bagEarth*	*glmboost*	*svmPoly*	*stepLDA*	**ScaledDown**
**AUROC**	0.85	0.86	0.82	0.79	0.75	0.81
**Accuracy**	1.00	0.98	0.88	0.89	0.73	0.90
**Sensitivity**	1.00	0.99	0.87	0.91	0.58	0.87
**Specificity**	1.00	0.98	0.89	0.88	0.88	0.93
**PPV**	1.00	0.98	0.89	0.88	0.83	0.92
**NPV**	1.00	0.99	0.87	0.91	0.68	0.89
**FPR**	0.00	0.02	0.11	0.12	0.17	0.08
**F1-score**	1.00	0.98	0.88	0.90	0.68	0.89
**MCC**	1.00	0.95	0.71	0.73	0.46	0.77

Figure SI-1. Mean value of AUROC, TPR and TNR metrics for the PCA, PCAUp, PCADown Scaled, ScaledUp and ScaledDown pre-processing conditions on the left panel. Right panels are the box-plots of the same metrics over the 5 clusters.

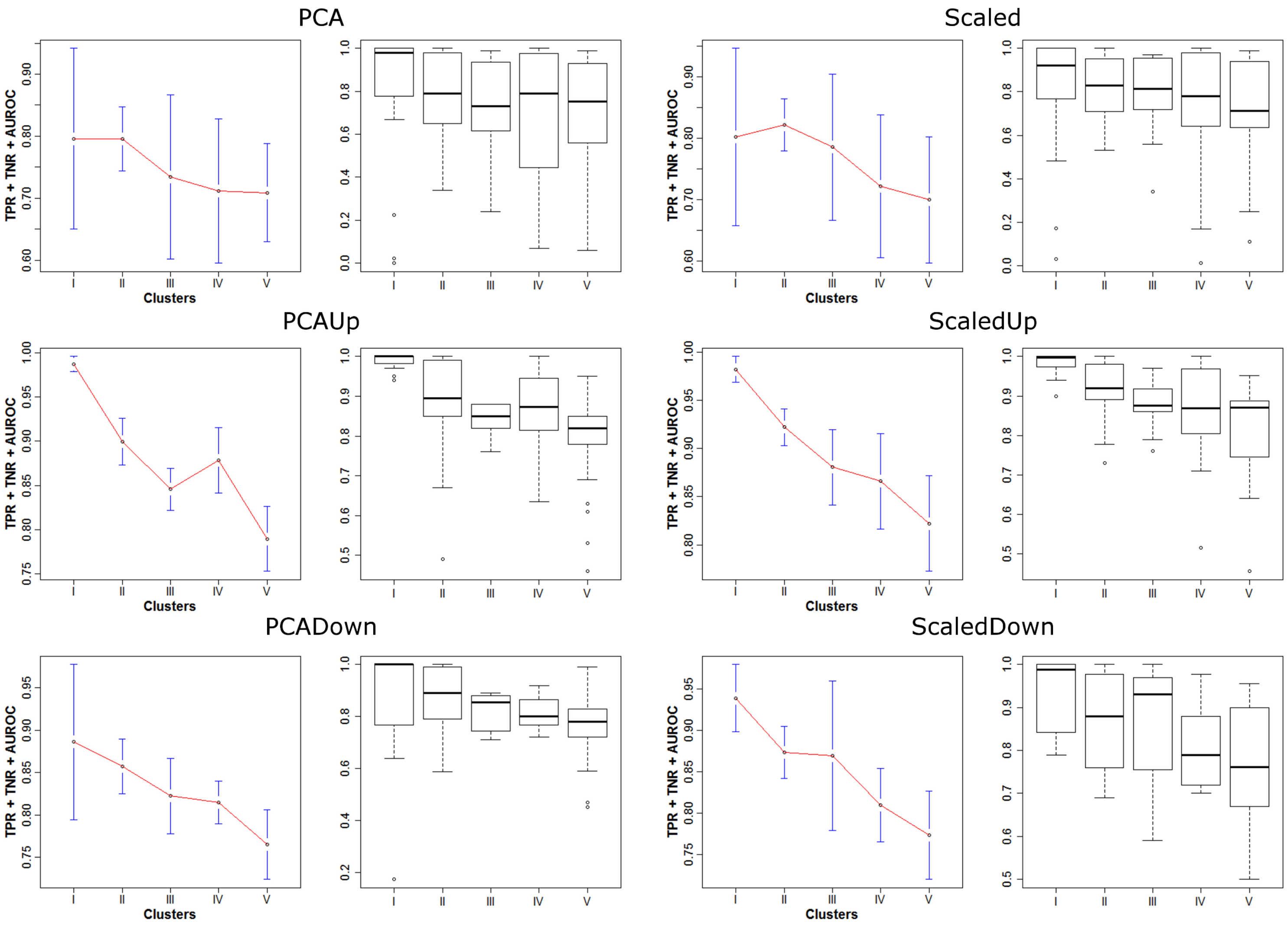

Figure SI-2. Training ROC curves of the best performing methods for each cluster (rf, ORFsvm, plr, svmPoly and pda) for the best pre-processing condition (ScaledUp). Sensitivity and specificity for the same methods are plotted on the bottom right corner, to provide a clearer representation of these values.

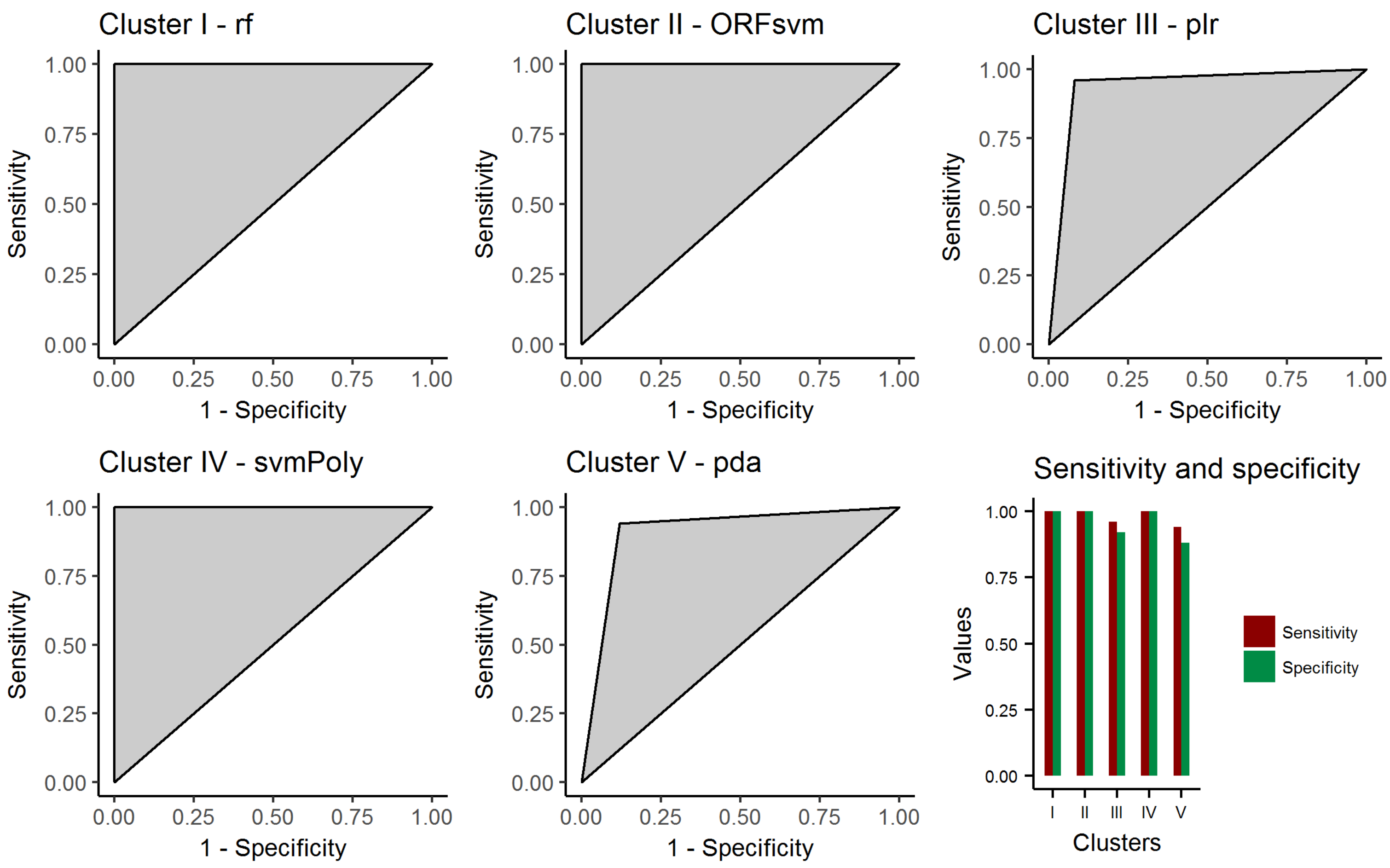

Figure SI-3. Screenshot of the submission page of the SpotOn webserver.

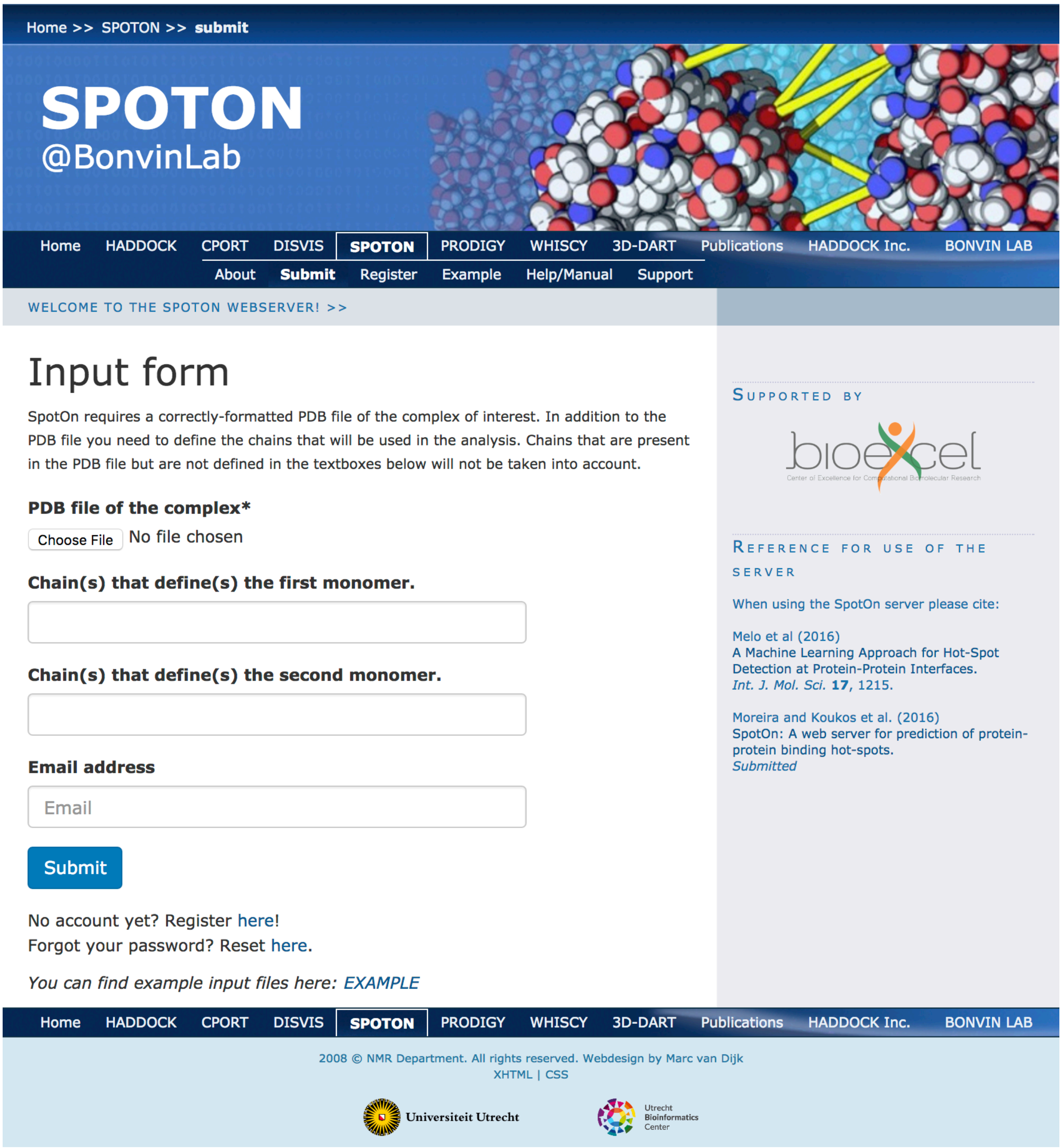

SPOTON DATASET

This document describes in a table all features used to train the SpotOn algorithm. The table is divided into column number/range, a brief description of each feature/group of features (Description) and the associated reference.

COLUMN NUMBER/RANGE	DESCRIPTION	REFERENCE
**1**	Protein Data Bank entry	[1]
**2:4**	Chain, residues number and name	
**5**	Experimental free binding energy difference (ΔΔG)/classification	[2-5]
**6**	Experimental HS/NS classification (residues with ΔΔG > 2.0kcal/mol are considered HS; all other are considered NS)	
**7:18**	Solvent Accessible Surface Area values	[6,7]
**19,20**	Number of amino acids at a given distance (2.5 and 4.0 angstrom, respectively) from the residue considered	[8]
**21**	Number of nearby hydrophobic residues	[8]
**22**	Total ΔSASA	[6,7]
**23:42**	Number of interfacial residues by chain	[8]
**43:82**	Post scoring substitution matrix (PSSM) values for each amino acid	[9,10]
**83:162**	Amphiphilic Pseudo-Amino Acid Composition (features extracted with protr module from R software)	[11]
**163:182**	Amino Acid Composition (features extracted with protr module from R software)	[11]
**183:886**	Scales-based descriptors derived by 20+ classes of 2D and 3D descriptors, including topological chemical descriptors, weighted holistic invariant molecular descriptors, vectors of hydrophobic, steric, and electronic properties, among others [12] (features extracted with protr module from R software)	[11]

REFERENCES

1 Berman, H. M. *et al.* The Protein Data Bank. *Nucleic acids research* **28**, 235-242 (2000).

2 Thorn, K. S. & Bogan, A. A. ASEdb: a database of alanine mutations and their effects on the free energy of binding in protein interactions. *Bioinformatics (Oxford, England)* **17**, 284-285 (2001).

3 Fischer, T. B. *et al.* The binding interface database (BID): a compilation of amino acid hot spots in protein interfaces. *Bioinformatics (Oxford, England)* **19**, 1453-1454 (2003).

4 Kumar, M. D. & Gromiha, M. M. PINT: Protein-protein Interactions Thermodynamic Database. *Nucleic Acids Res* **34**, D195-198, doi:10.1093/nar/gkj017 (2006).

5 Moal, I. H. & Fernandez-Recio, J. SKEMPI: a Structural Kinetic and Energetic database of Mutant Protein Interactions and its use in empirical models. *Bioinformatics (Oxford, England)* **28**, 2600-2607, doi:10.1093/bioinformatics/bts489 (2012).

6 Martins, J. M., Ramos, R. M., Pimenta, A. C. & Moreira, I. S. Solvent-accessible surface area: How well can be applied to hot-spot detection? *Proteins* **82**, 479-490, doi:10.1002/prot.24413 (2014).

7 Melo, R. *et al.* A Machine Learning Approach for Hot-Spot Detection at Protein-Protein Interfaces. *International journal of molecular sciences* **17**, doi:10.3390/ijms17081215 (2016).

8 Humphrey, W., Dalke, A. & Schulten, K. VMD: Visual molecular dynamics. *Journal of Molecular Graphics* **14**, 33-38, doi:http://dx.doi.org/10.1016/0263-7855(96)00018-5 (1996).

9 Altschul, S. F., Gish, W., Miller, W., Myers, E. W. & Lipman, D. J. Basic local alignment search tool. *J Mol Biol* **215**, 403-410, doi:10.1016/S0022-2836(05)80360-2 (1990).

10 Pearson, W. R. BLAST and FASTA similarity searching for multiple sequence alignment. *Methods Mol Biol* **1079**, 75-101, doi:10.1007/978-1-62703-646-7_5 (2014).

11 Xiao, N., Cao, D. S., Zhu, M. F. & Xu, Q. S. protr/ProtrWeb: R package and web server for generating various numerical representation schemes of protein sequences. *Bioinformatics (Oxford, England)* **31**, 1857-1859, doi:10.1093/bioinformatics/btv042 (2015).

12 Ivanciuc, O. Chemical graphs, molecular matrices and topological indices in chemoinformatics and quantitative structure-activity relationships. *Current computer-aided drug design* **9**, 153-163 (2013).
